# Obesity Prevention and Reduction in China Using the Social Media Platform WeChat: Scoping Review

**DOI:** 10.2196/65538

**Published:** 2025-12-11

**Authors:** Yinuo Wang, Xuxiu Zhuang, Samantha Sundermeir, Joel Gittelsohn

**Affiliations:** 1 Department of Food Science and Nutrition, The Hong Kong Polytechnic University, Hong Kong, China (Hong Kong); 2 Institute of Health Sciences, China Medical University, Shenyang, China; 3 Center for Human Nutrition, Department of International Health, Johns Hopkins Bloomberg School of Public Health, 615 N Wolfe Street, Baltimore, MD, 21205, United States, 1 410-955-3927, 1 410-955-0196

**Keywords:** obesity, digital interventions, mHealth, WeChat, scoping review, prevention, public health, Chinese, PRISMA, Preferred Reporting Items for Systematic Reviews and Meta-Analyses

## Abstract

**Background:**

Digital interventions for obesity have demonstrated efficacy in obesity prevention and management. The emergence of smartphones and ubiquitous apps such as WeChat represents potential modality to enhance the reach, sustainability, and cost-effectiveness of such interventions. By the end of the first quarter of 2024, WeChat had approximately 1.36 billion monthly active users, accounting for 96.5% of China’s population. The use of this platform for obesity interventions has been validated in multiple Chinese trials, most published in Chinese language journals.

**Objective:**

We aim to synthesize the existing evidence on obesity interventions delivered through WeChat to generate implications for future intervention design and development, thereby reaching an international audience.

**Methods:**

We conducted a scoping review of PubMed and China National Knowledge Infrastructure using search terms including “WeChat,” “obesity,” “weight,” “BMI,” “waist circumference,” “hip circumference,” “waist-to-hip ratio,” “body fat,” “skin fold thickness,” and these Chinese equivalents “weixin,” “feipang,” “tizhong,” “tizhongzhishu,” “yaowei,” “tunwei,” “yaotunbi,” “tizhi,” and “pizhehoudu.” We included only original research studies, theses, or dissertations with measurable outcomes that used WeChat functions as intervention strategies. Study quality was assessed using the National Institutes of Health Quality Assessment Tool, with specific tools selected based on study design. Descriptive statistics were applied, with categorical variables summarized as frequencies and percentages (n, %) to report study distribution.

**Results:**

Our scoping review based on PubMed and China National Knowledge Infrastructure identified 665 initial records, among which 43 studies met eligibility criteria and were included for data extraction to characterize intervention details. Results indicated effectiveness in 86.0% (37/43) of studies, with WeChat-assisted obesity interventions achieving significant short- and long-term weight loss measured by objective outcomes (body weight, BMI, waist circumference, hip circumference, waist-to-hip ratio, and body fat percentage). However, formative research informing intervention design was insufficient. Common methodological limitations included lack of randomization and blinding (42/43, 97.7%) and unreported intervention compliance metrics (39/43, 92.0%). Functionally, interventions primarily used “WeChat group” and “Official Account”—public accounts that provide health education, diet or physical activity logging, and other features.

**Conclusions:**

Overall, WeChat represents a promising platform for obesity interventions; however, current apps fail to leverage its full features (eg, online payment and live streaming). Key limitations include methodological heterogeneity and cultural specificity, which were addressed through narrative synthesis stratified by study types. Future research should incorporate the formative phase and use more rigorous methodologies such as randomized controlled trials to optimize intervention design and delivery via this modality.

## Introduction

Obesity is a global health crisis affecting low- and middle-income as well as high-income countries [1,2]. According to the 2023 Chinese Nutrition and Chronic Diseases Survey, more than 50% of Chinese adults were overweight or obese [3], with prevalence rates of nearly 20% among adolescents and 10% in children younger than 6 years [3]. Obesity is strongly associated with noncommunicable chronic diseases (NCDs), particularly fatty liver, diabetes, hypertension, and dyslipidemia [4]. Furthermore, NCDs accounted for 90.1% (9.6 million) of deaths and 84.9% (324.6 million) of disability-adjusted life years in China in 2019 [5]. Wang et al [6] project that the prevalence of overweight or obesity in Chinese adults will reach 65.3% (790.0 million) by 2030, potentially leading to a 22.0% (more than CNY 400.0 billion [more than US $60 billion]) increase in total medical costs. In response, the Healthy China Action Plan 2019‐2030 established 15 key strategies, including 11 focused on NCDs intervention [7]. Thus, implementing evidence-based obesity interventions is critical for preventing future NCD-related mortality.

Although nearly 90% of countries globally have committed to addressing obesity [8], the efficacy of interventions for sustained weight control remains limited [9,10]. Social media platforms are increasingly used in obesity interventions, particularly for health education delivery [11,12]. Digital health apps can expand geographical reach and overcome barriers to in-person care [13], as demonstrated during the COVID-19 pandemic when obesity interventions rapidly shifted toward mobile health (mHealth) solutions [14]. In the United States, of more than 100,000 available health-related apps, more than 28,000 target weight management [15]. By late 2023, China had 1.09 billion web users (77.5% penetration) [16], with web-based health care users reaching 413.9 million—a 14.2% annual increase [16]. By reducing medical expenses, improving adherence, and promoting communication between researchers and participants [17,18], mHealth enables cost-effective and individualized interventions [19,20]. According to a systematic review about weight management mobile technology, there are a great number of apps designed to facilitate obesity interventions [21]. For example, Oviva’s NHS Digital–approved app is a convenient way to track weight, keep a food diary, and deliver dietary guidance [22-25]; Kurbo is an app designed to help teenagers and children aged 8-17 years to lose weight, using the Traffic Light System designed by Stanford University [26-28]; W8Loss2Go (Weight Loss to Go), implementing a 3-phase approach (identifying problem foods, minimizing eating between meals, and gradually reducing portion sizes), is effective for weight loss [29]; Weight Watchers Online is a weight loss app that focuses on food, physical activity (PA), sleep, and mindset [30,31]; and BeHealthy provides education on nutrients, sleep, and yoga [32]. While designed for weight management guidance and monitoring, few apps incorporate health care professional input or rigorous validation [33].

**Figure 1.**
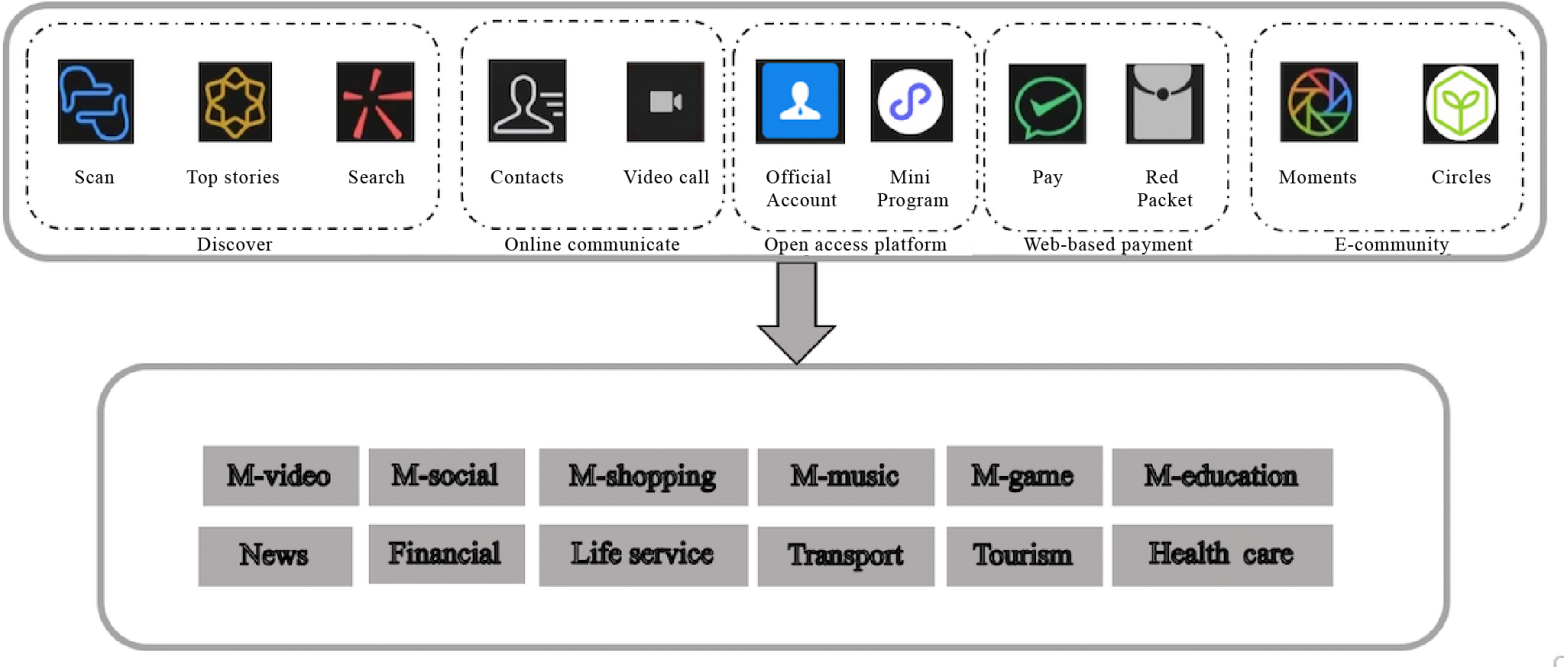
Functions of the WeChat platform.

WeChat, a dominant media platform with multiple functions launched by the Tencent Company in China on January 21, 2011, reached 1.359 billion monthly active users in Q1 2024—a 3% increase from March 2023 [34]. Monthly active user, a key metric reflecting monthly engagement with WeChat services, demonstrates its pervasive adoption. Although initially the primary function of WeChat was chatting, with version updates, WeChat has updated more and more features integrating social networking, mobile payment, and enterprise solutions through features such as Official Account for content dissemination, Mini Program for lightweight apps, and WeChat group for real-time group communication (Figure 1). The platform’s multifunctional ecosystem facilitates diverse aspects of users’ lives (Figure 1), including health education delivery [35] and chronic disease management [36] such as hypertension [37,38] and diabetes [39-42].

Although previous studies have summarized the efficacy of mHealth apps for weight management, none have systematically detailed the functional implementation of WeChat in obesity interventions or established evidence-based frameworks for optimizing these functions in future projects. The goal of this study was to conduct a scoping review of existing obesity intervention studies through the WeChat platform and its potential use in future research. We focused on answering the following questions: (1) What WeChat functions have been used in existing obesity prevention or management intervention studies?; (2) What are the primary methodological limitations in these studies?; (3) How can future WeChat-delivered interventions be optimized?

## Methods

### Search Strategy

To identify literature on WeChat-based obesity interventions up to April 14, 2024, we searched PubMed and China National Knowledge Infrastructure (CNKI, the largest academic database, aggregating more than 95% of officially published Chinese scholarly resources), using the following search terms: “WeChat,” “obesity,” “weight,” “BMI,” “waist circumference (WC),” “hip circumference (HC),” “waist-to-hip ratio (WHR),” “body fat (BF),” “skin fold thickness,” and Chinese equivalent characters: “weixin,” “feipang,” “tizhong,” “tizhongzhishu,” “yaowei,” “tunwei,” “yaotunbi,” “tizhi,” and “pizhehoudu” (Table S1 in Multimedia Appendix 1).

### Study Selection

After eliminating duplicates and off-topic studies, all the papers that described WeChat-based obesity interventions were screened against the following inclusion criteria: (1) full-text original or empirical human research published in peer-reviewed journals (including trials with or without control groups, and blinded or nonblinded designs); (2) WeChat as primary intervention delivery platform (eg, dietary or PA guidance, dietary or PA diary logging, etc); (3) Chinese degree theses or dissertations from the CNKI dataset; and (4) objective anthropometric outcomes including body weight, BMI, WC, HC, WHR, body fat percentage (BFP), or skin fold thickness. The exclusion criteria included (1) studies using WeChat solely for participant recruitment or follow-up; (2) self-reported anthropometric parameters; and (3) study protocols, animal studies, reviews, patents, books, or inaccessible texts. This process was done by 2 authors (YNW and XXZ) who independently screened titles and abstracts and conducted full-text reviews to identify the final set of eligible papers for inclusion. Any conflicts during this process were resolved through team consensus discussions. The flow diagram was adapted from the official PRISMA (Preferred Reporting Items for Systematic Reviews and Meta-Analyses) template with modifications specific to our search strategy [43].

### Data Extraction

The following data were extracted from each study: authors, sample size, presence of control groups, WeChat functions used, interventions duration, outcomes measured, and intervention effectiveness. Intervention effectiveness was categorized as (1) “effective” if all predefined weight-related outcomes (eg, BMI, weight, WC, HC, WHC, BFP, etc) showed statistically significant improvements; (2) “partially effective” if only a subset of predefined outcomes demonstrated significant positive changes; and (3) “not” if none of the predefined outcomes demonstrated significant changes. Themes were synthesized from tile or abstract review followed by full-text review using a blinded, independent dual review process by 2 of the authors (YNW and XXZ). Conflicts were resolved through discussion, with a third author (JG) who would arbitrate unresolved conflicts.

### Quality Assessment

A detailed quality assessment was conducted using the Quality Assessment Tool for Controlled Intervention Studies and Before-After (Pre-Post) Studies With No Control Group designed by National Institutes of Health (NIH) [44]. The assessment tool of controlled intervention studies is applicable to those with control group and intervention strategies, containing 14 items to evaluate the research. Before-after studies refer to those without control groups, instead comparing outcomes of baseline and the end. The tool for before-after studies consists of 12 items. All scoring criteria were evaluated as “yes,” “no,” “not applicable (NA),” or “not reported (NR).” For each item, we assigned 1 point for “yes” and “NA,” zero points for “no” and “NR.” According to the total score, studies were further rated as “good” (10 for controlled intervention studies; 9 for before-after studies), “fair” (5-9 for controlled intervention studies; 5-8 for before-after studies), and “poor” (zero to 4 for both). Two authors (YNW and XXZ) independently completed the quality assessment for each study. Conflicts were resolved via discussion, with a third author (JG) arbitrating unresolved cases.

### Statistical Analysis

Statistical analyses were performed using GraphPad Prism 8.4.0 (GraphPad). Descriptive statistics were applied, with categorical variables summarized as frequencies and percentages (n, %) to report study distribution across classifications, as well as the quality assessment outcomes of included studies.

## Results

### Overview

**Figure 2.**
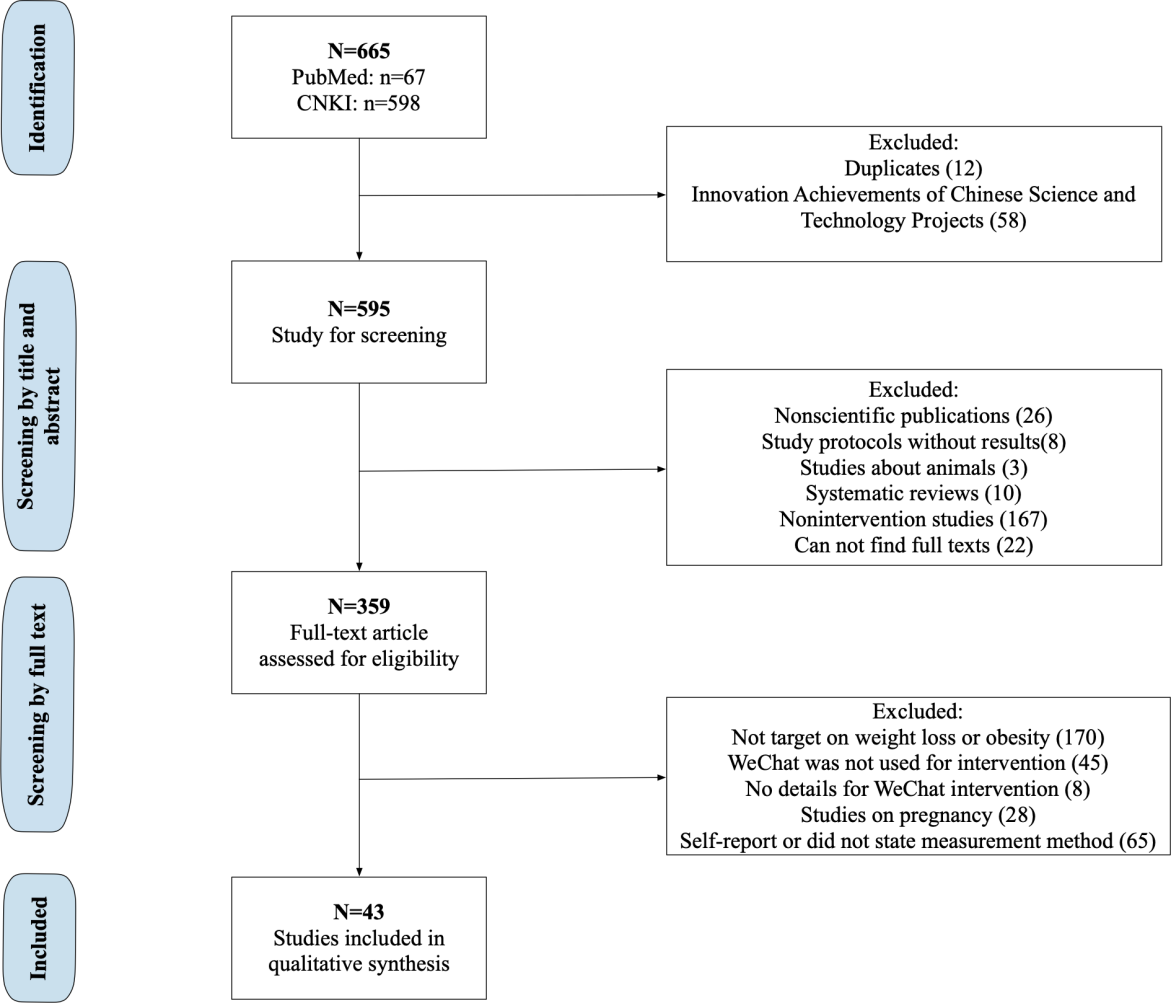
Flow diagram detailing the study selection process. CNKI: China National Knowledge Infrastructure.

From the initial 665 papers (67 from PubMed and 598 from CNKI), 12 duplicates and 58 Innovation Achievements of Chinese Science and Technology Project designs were excluded. During the first round of title or abstract review, 236 publications were excluded, in which 26 of them were nonscientific publications, 8 study protocols, 3 animal studies, 10 systematic reviews, 167 nonintervention studies, and 22 conference abstracts. Of the 359 studies undergoing full-text review, 315 (87.7%) were excluded due to the following reasons: focus beyond weight loss or obesity (170, 54.0%), WeChat not used for intervention delivery (45, 14.3%), inadequate WeChat intervention strategy details (8, 2.5%), pregnancy-focused studies (28, 8.9%), and self-reported or unverified measures (65, 20.6%). The flow diagram is shown in Figure 2. Eight (18.6%) studies [45-52] were in English with information about study population, control group presence, intervention methods, intervention duration, health outcomes, and intervention effectiveness summarized in Table 1. A total of 35 (35/43, 81.4%) studies [53-87] were in Chinese with study details shown in Table 2. Among the 43 studies finally included, 20 (46.5%) [53,57,59-63,65,70,72,74-76,79,81,83-87] of them were theses or dissertations. All the studies were conducted in mainland China. None of the papers reported using formative research for intervention development. Sample sizes ranged from 20 [64] to 15,310 [46]. Among the 43 papers, 33 (76.7%) [45-47,49-51,56-63,65-67,70-72,74-76,78-87] included a control group, and 26 (60.5%) [45,47,49-51,56-62,65,66,70,72,74,76,78-80,82-86] reported assigning participants randomly. The duration of interventions spanned 4 weeks [51] to 36 months [52].

**Table 1.** Summary of studies using WeChat for obesity interventions in English (in order of publication date; n=8).

Authors	Sample	Control group	Intervention related to WeChat	Duration	Outcome	Effective^a^ or not
Li et al [49]	42 full-time postgraduate students, Shanghai	Yes (randomized)	WeChat: submit PA^b^ diaries WeChat group: connect with other participants for social support	1 month	BW^c^, BFP^d^, WHR^e^, and BMI	Partly effective: BFP
Chen et al [47]	102 women from 2 community health centers in Changsha	Yes (randomized)	WeChat: set up fruit or vegetable intake goals and daily steps, educational modules along with tailored tips and messages, and messages to encourage and reinforce positive behavioral changes	6 months	WC^f^ and BMI	Not
Han et al [50]	120 overweight and obese patients with T2DM^g^ from a hospital in Nanjing	Yes (randomized)	Official Account: information (video teaching, compulsory course, and Monday paper); function (diet form, resistance or aerobic exercise, body measurement, and homework feedback)	12 months	BMI, WC, HC^h^, WHR, and BFP	Partly effective: BMI
Xia et al [45]	343 participants with T2DM	Yes (randomized)	WeChat group: post BG test result, completion of exercises, medication, self-management data WeChat voice call: weekly and monthly summaries to participants	6 months	BW and BMI	Partly effective: BW
Yang et al [52]	977 overweight patients from a hospital in Zhejiang	No (before-after)	WeChat group: report anthropometry (eg, body weight), daily dietary intakes, and PA Mini Program: report body weight WeChat: personal feedback on weight management	36 months	BW	Effective
Ling et al [51]	300 inpatients with primary hypertension in a hospital in Xi’an	Yes (randomized)	Official Account: posthypertension-related knowledge WeChat group: posthypertension-related knowledge; distribute information about educational lectures One-to-one chatting: answer questions	1 month	BMI and WC	Not
Liu et al [48]	300 overweight or obese participants in Sichuan	No (before-after)	WeChat: postdinner plates and beverage	3 months	BW, BMI, WC, BFP, and WHR	Effective
He et al [46]	15,310 occupational participants in the Shunyi District of Beijing	Yes (those did not follow the Official Account)	Official Account: Health education including intervention of PA and diet	6 months	BW	Partly effective: males

a There were favorable changes in the indices after intervention according to their own reports in the study.

b PA: physical activity.

c BW: body weight.

d BFP: body fat percentage.

e WHR: waist-to-hip ratio.

f WC: waist circumference.

g T2DM: type 2 diabetes mellitus.

h HC: hip circumference.

**Table 2.** Summary of studies using WeChat for obesity interventions in Chinese (in order of publication date; N=35).

Authors	Sample size	Control group	Intervention related to WeChat	Duration	Outcome	Effective^a^ or not
Yinbao^b^ [84]	1840 from the 51st Regiment of the Third Division in Xinjiang	Yes (randomized)	Official account: post health knowledge	12 months	BW^c^, WC^d^, HC^e^, and BMI	Partly effective: HC and BMI
Lu^b^ [83]	62 patients with coronary heart disease in a hospital in Tangshan	Yes (randomized)	Mini Program: record diets WeChat group: supervise diet, share educational material, and healthy diet competitions WeChat live streaming: educational lectures	3 months	BW, BMI, and WC	Partly effective: BW and BMI
Youxuan^b^ [74]	295 people at high risk of hypertension from 15 communities in Haikou	Yes (randomized)	WeChat group: reminding to upload diet and PA^f^, upload health education, and health guidance	12 months	BMI, WC, and HC	Effective
Jun^b^ [86]	60 patients with prediabetes aged 50‐70 years from a health management center, Guangzhou	Yes (randomized)	WeChat group: share heart rate data, remind to practice, and communication to promote PA	3 months	BW and BFP^g^	Effective
Linjuan^b^ [53]	165 patients with T2DM^h^ from a hospital in Inner Mongolia	No (before-after)	Mini Program: record glucose, evaluate diet, daily step recording, nutrition recipe, health knowledge, and remind medicine intake	6 months	BMI	Effective
Tianmeng et al [69]	1292 residents with higher blood pressure in Shandong	No (before-after)	WeChat group: post poster or papers about health knowledge; answer daily questions	3 months (100 days)	WC, HC, BW, BMI, and BFP	Effective
Mengqin^b^ [76]	480 patients with essential hypertension aged 18–70 years from 5 towns in rural areas of Shanxi	Yes (randomized)	WeChat group: post health knowledge of posters, papers, and videos	3 months	BMI, WC	Not
Wei et al [78]	200 students with normal blood pressure enrolled in 2017 from a university in Xizang	Yes (randomized)	WeChat group: post health education of essays, cartoon, pictures, videos One-to-one chatting: provide consultancy	6 months	BMI	Effective
Ruixue et ali [73]	63 patients with overweight or obesity from a hospital in Chongqing	No (before-after)	WeChat group: post health knowledge, upload personal PA recording, communications among participants, and upload daily diet recording and evaluated by physicians	3 months	BW, BMI, BFP, and WC	Effective
Lu [83]	152 patients with diabetes from 5 communities in Shenzhen	Yes (randomized)	WeChat group: post health knowledge, discussion, and evaluation on nutrition	3 months	BMI and WC	Effective
Xiaoyuan et al [58]	100 patients with phlegm dampness constitution coronary heart disease from a hospital in Nanning	Yes (randomized)	WeRun: record daily steps WeChat group: supervise daily diet and PA	6 months	BW, WC, and BMI	Effective
Zihao et al [54]	54 overweight or obese people from a hospital in Guizhou	No (before-after)	WeChat group: supervise and provide guidance for daily diets and PA; answer questions of participants	3 months	BW, WC, HC, and BFP	Effective
Weiwei^b^ [81]	68 older adult patients with chronic diseases with physical inactivity in 2 communities in Bengbu	Yes (participants from Hubin)	WeChat group: post health education of materials, upload weekly PA recording, and health guidance	6 months	BMI and WHR^i^	Effective
Jinhua^b^ [59]	107 older adult patients with hypertension from 4 communities in Changsha	Yes (randomized)	WeChat group: share healthy diet and PA videos, upload healthy knowledge about hypertension, and web-based consultancy	6 months	BMI and WC	Partly effective: BMI
Peijun [65]	120 patients with obesity who underwent physical examination from Sichuan University	Yes (randomized)	WeChat group: post health education materials One-to-one chatting: answer questions Official Account: Traditional Chinese Medicine Health Preservation Special Issue	6 months	BW, BMI, BFP, and WHR	Effective
Jielili^b^ [75]	48 cases of obesity from Jinglao Community, Hetian	Yes (obesity and normal weight control)	WeChat group: health education; psychological cognition, and weight management	3 months	BW, WC, and HC	Effective
Ziwei^b^ [70]	40 young and middle-aged patients with hypertension from 3 community health care centers in Hengyang	Yes (randomized)	WeChat group: upload health education, share BMI and WC change to encourage each other, supervise daily steps, guidance on smoking and drinking, remind on medicine intake, and share music and videos to relax	6 months	BMI and WC	Partly effective: BMI
Yuepeng^b^ [61]	40 patients with hyperuricemia in a Tianjin hospital	Yes (randomized)	Official Account: education on diet, PA, and medication knowledge WeChat group: post health education contents, share videos of PA, and answer questions	6 months	BW, WC, HC, BMI, and WHR	Not
Xi et al [55]	47 employees in an affiliated hospital	No (before-after)	WeChat group: share pictures of daily diets and PA; one-to-one dietary guidance by professional nutritionist (including intervention of PA and diet)	1.5 months	BW, BMI, BF, BFP, and WC	Effective
Jianxin et al [71]	130 older adult hypertensive patients from Chinese PLA Hospital No. 305	Yes (not randomized, grouped by first clinic date)	Mini Program: diagnosis and treatment, medication guidance, and data management	6 months	BW, BMI, WC, WHR, and BFP	Partly effective except BW
Li^b^ [62]	100 patients with T2DM from community health service center in Chongqing	Yes (randomized)	Official Account: guidance on personal diet and PAs; upload situation of dietary, PA, glucose, and blood pressure; and supervision and health education	12 months	BW, BMI, WC, HC, and BFP	Effective
Yingming^b^ [63]	60 patients with overweight or obesity and polycystic ovary syndrome from 2 hospitals in Hainan	Yes (not randomized, grouped by first clinic date)	WeChat group: experience sharing, life behavior guidance, and supervision	3 months	BW, BMI, WC, HC, and WHR	Partly effective except HC
Lijuan^b^ [85]	100 young and middle-aged patients with hypertension from all community health service centers in Fuding	Yes (randomized)	WeChat group: health education, consultancy by a doctor, and remind of changing unhealthy timetable	6 months	BW, WC, HC, and BMI	Not
Jiawei^b^ [60]	128 patients with T2DM treated or hospitalized in a hospital, Chengdu	Yes (randomly)	Official Account: post intervention strategies, collect information WeChat group: share intervention strategies	3 months	WHR	Effective
Yuexi et al [66]	60 residents with prediabetes from a hospital in Nanning	Yes (randomly)	WeChat group: upload health knowledge, answer questions	24 months	WC and BW	Effective
Jingxia et al ^b^ [87]	278 employees from 4 worksites in Yang River Delta	Yes (randomly select 2 worksites)	Official Account: post information about obesity, diet and PA, querying energy of food, querying PAs, web-based competition on daily diet, and PAs with rewards	12 months	BW, BMI, WC, HC, and WHR	Partly effective: BW, BMI, and HC
Rongrong ^b^ [79]	314 individuals with prediabetes diagnosed by a hospital in Shandong	Yes (randomly)	Official Account: health education WeRun: supervise daily steps WeChat group: health lectures and health educations in voice, words, and pictures	7.5 months	BW, BMI, WC, and HC	Effective
Xujuan et al [77]	36 people with obesity from a physical examination center in Taiyuan	No (before-after)	WeChat group: exercise prescription	2 months	WC, BW, BMI, and BFP	Effective
Xiao and Dafang [68]	301 volunteers	No (before-after)	WeChat group: provide guidance on diet, PAs, and psychology	1 month	BW	Effective
Xiaojuan et al [64]	20 overweight patients with prediabetes from a hospital in Wuxi	No (before-after)	WeChat group: upload health knowledge, share daily food and energy intake, and answer questions	6 months	BW, BMI, and WC	Effective
Bei^b^ [57]	262 middle-aged and young patients with primary hypertension from a community health service center in Urumqi	Yes (randomly)	WeChat group: upload health education material weekly, answer questions during intervention, and communicate with other participants	6 months	BMI	Not
Huirong et al [80]	223 patients with chronic noninfectious diseases from a hospital in Xinjiang	Yes (Randomly)	Official Account: Public Health Microworld	3 months	BW, BMI, WC, HC, WHR	Effective
Di ^b^ [72]	40 obesity children aged 10‐11 from one of primary school in Beijing	Yes (randomly)	WeChat group: share pictures of 3 meals of children; upload web-based education materials (including PA and diet)	2 months	BW, BMI, WC, HC, and BFP	Partly effective: BW and BMI
Jiangping et al [67]	77 college teachers from 2 universities in Harbin	Yes (1 university)	WeChat group: upload health education and share PAs videos	3 months	BMI and WC	Effective
Dongmei et al [56]	86 coronary heart disease patients from a hospital in Shandong	Yes (Randomly)	WeChat group: upload health education, supervise participants to follow personal treatment, and communicate with others	12 months	BMI	Effective

a There were favorable changes in the indices after intervention according to their own reports in the study.

b Thesis for degree (master's and PhD).

c BW: body weight.

d WC: waist circumference.

e HC: hip circumference.

f PA: physical activity.

g BFP: body fat percentage.

h T2DM: type 2 diabetes mellitus.

i WHR: waist-to-hip ratio.

### Methods of Intervention

Among the 43 papers ultimately included, 32 (74.4%) [46-48,50,53-57,59,62-64,66-77,80-82,84-87] used a single WeChat function for interventions, while 11 (25.6%) [45,49,51,52,58,60,61,65,78,79,83] used multiple functions of WeChat to implement their intervention studies. “WeChat group” and “Official Account” were 2 functions used the most, with 33 (76.7%) [45,49,51,52,54-61,63-70,72-79,81-83,85,86] and 11 (25.6%) [46,50,51,60-62,65,79,80,84,87] studies using them, respectively (Table 3). WeChat groups primarily facilitated communication between investigators and participants. Official Accounts were mainly used to deliver dietary or PA education and record daily diets and PA. Four (9.3%) studies [52,53,71,83] used Mini Program in their intervention strategies with similar but expanded functions as Official Account. Three (7.0%) studies [51,65,78] used one-to-one chatting functions enabling confidential consultations. In addition, 2 (4.7%) studies [58,79] used WeRun to conduct interventions. Meanwhile, another 2 used voice call [45] and live streaming [83] separately. A total of 4 (9.3%) studies did not clearly report the specific functions of WeChat they used in their intervention (refer to “Intervention” item in Tables1  2 for more details).

**Table 3.** WeChat components used in the interventions.

Function	Definition	Values, n (%)
WeChat Group	Containing up to 500 members; all the members can share information and chat in the group; daily attendance of web-based activities	33 (76.7)
Official Account (accounts that developers or merchants apply for on the WeChat official platform)	The platform uses communication and interaction with specific groups of text, pictures, voice, and video, forming a mainstream online and offline WeChat interactive marketing Way	11 (25.6)
Mini Program	Lightweight apps that run within the WeChat environment; smaller than dedicated apps; no need to download	4 (9.3)
One-to-One Chatting	Sending text messages and voice messages; making video calls and voice calls; sharing pictures or WeChat public press paper; transferring money to WeChat friends	3 (7.0)
WeRun	Recording the number of daily steps taken in walking	2 (4.7)
Voice Call	Free communicating method with your WeChat account followers under network environment	1 (2.3)
WeChat Live Streaming	Directly get access to live streams within WeChat without additional download; providing real-time interaction, content sharing, and more	1 (2.3)
Not specified	Did not mention which specific functions were used for intervention	4 (9.3)

### Descriptive Statistics on Intervention Efficacy

In summary, 25 out of 43 (58.1%) studies [48,52-56,58,60,62,64-69,73-75,77-82,86] demonstrated significant improvement across all obesity-related measures. Of the studies remaining, 12 (27.9%) [45,46,49,50,59,63,70-72,83,84,87] showed partial effectiveness on the selected measures and 6 (14.0%) [47,51,57,61,78,85] reported no significant effects with a duration period no longer than 6 months. BMI was the most commonly used outcome measure for obesity, in which 27 (77.1%) [48,50,53,55,56,58,59,62-65,67,69-74,77-84,87] out of 35 [45,47-51,53,55-59,61-65,67,69-74,76-85,87] were significantly decreased after intervention. A total of 28 (65.1%) studies [45,46,48,49,52,54,55,58,61-66,68,69,71-73,75,77,79,80,83-87] assessed body weight, in which 23 (82.1%) [45,46,48,52,54,55,58,62-66,68,69,72,73,75,77,79,80,83,86,87] showed weight loss after intervention. A total of 30 (69.8%) studies [47,48,50,51,54,55,58,59,61-64,66,67,69-77,79,80,82-85,87] assessed WC as outcome, in which 18 (60.0%) [48,54,55,58,62-64,66,67,69,71,73-75,77,79,80,82] of them showed a significant reduction after intervention. For HC, a total of 14 (32.6%) studies [50,54,61-63,69,72,74,75,79,80,84,85,87] were considered it as outcome, in which 9 (64.3%) [54,62,69,74,75,79,80,84,87] showed significant decrease. Similarly, a total of 11 (25.6%) studies [48-50,60,61,63,65,71,80,81,87] assessed WHR as outcome, in which 7 (63.6%) [48,60,63,65,71,80,81] of them showed a significant decrease at the end of the study. Of the 14 (32.6%) studies [48-50,54,55,62,65,69,71-73,77,86] assessing BF, only 1 (7.1%) [55] of them used BF and all others used BFP. BF and BFP were shown to be impacted mostly by interventions compared with other measures in these studies because 12 (85.7%) [48,49,54,55,62,65,69,71,73,77,86] out of 14 [48-50,54,55,62,65,69,71-73,77,86] showed that they were significantly decreased (Tables1  2).

### Quality Assessment of Study

Using the NIH Study Quality Assessment Tool, we assessed a total of 33 studies [45-47,49-51,56-63,65-67,70-72,74-76,78-87] that had a control group, with 4 (12.1%) [50,70,76,79] rated as good quality, 25 (75.8%) [45,47,49,51,56-63,65-67,71,74,78,81-87] as fair quality, and 4 (12.1%) [46,72,75,80] as poor (See Table S2 in Multimedia Appendix 1). The number of studies meeting each assessment item is shown in Table 4. Twenty-seven (81.8%) [45,47,49-51,56-63,65,66,70,72,74-76,78,79,82-86] of them were described as randomized, in which 16 (59.3%) [50,51,56-60,62,65,66,70,74,76,79,83,85] reported randomization methods that were regarded as adequate (see Table S2 in Multimedia Appendix 1, item 11 and item 22). Only 2 (6.1%) studies [50,76] reported using a double-blinded randomization process, and only 1 (3.0%) study [50] reported that outcome assessors were blinded. Among all the studies with control groups, 6 (18.2%) [49,72,78,80,82,86] did not report demographic information separately for intervention and control groups, and 4 (12.1%) studies [46,66,75,87] reported significant differences in some demographic or other factors related to the outcome being assessed (see Table S2 in Multimedia Appendix 1, item 6). Two (6.1%) studies [45,87] reported an overall dropout rate exceeding 20%, with exact values of 23.1% [45] and 28.4% [87] separately, and 4 (12.1%) studies [65,67,72,75] provided no information about dropout rate (see Table S2 in Multimedia Appendix 1, item 7). In addition, 3 studies reported high intervention adherence [47,72,86], while 3 others reported low adherence [46,61,87]. Besides, intent-to-treat analysis was used in data analysis in 3 studies to avoid bias caused by dropout [47,50,87].

A total of 10 out of 43 (23.3%) studies [48,52-55,64,68,69,73,77] used a before-after design without a control group. According to the rating, all of them [48,52-55,64,68,69,73,77] were rated as fair quality (Table S3 in Multimedia Appendix 1). The number of studies meeting each assessment item is shown in Table 5. All the before-after studies [48,52-55,64,68,69,73,77] stated research questions or objectives clearly and presented specific inclusion and exclusion criteria. Two (20.0%) studies [48,52] included participants who could represent the population who would be eligible, and 1 (10.0%) study [69] enrolled all the eligible participants meeting the entry criteria. Although some of the studies [48,53,54,64,73] provided information about sample size calculations and power to detect statistical differences, they claimed insufficient power as a limitation. All the studies [48,52-55,64,68,69,73,77] clearly and consistently described intervention strategies and used effective and objective outcome measurements. However, none reported whether blinding was used during intervention implementation or data analysis. All studies [48,52-55,64,68,69,73,77] prespecified outcome measures, which were applied clearly and consistently throughout the study process. Three studies reported a loss to follow-up rate exceeding 20%, with rates of 82.4% [52], 24.3% [53], and 55.5% [73], respectively. All studies [48,52-55,64,68,69,73,77] analyzed changes in outcomes of interest before and after the intervention and presented *P* values, but only 3 studies [52,64,68] measured outcomes multiple times before, during, and after the intervention. None of these studies was conducted at the group level; thus, it was not applicable to account for the use of individual-level data in determining group-level effects in the statistical analysis.

**Table 4.** Numbers of studies with control group that met each assessment item.

Assessment item	Values, n (%)
Outcomes valid and reliable	33 (100.0)
Outcome prespecified	33 (100.0)
Dropout rate between groups	29 (87.9)
Randomized controlled trial	27 (81.8)
Overall dropout rate	27 (81.8)
Groups similar	23 (69.7)
Randomization adequate	16 (48.5)
Treatment allocation concealed	15 (45.5)
Sample size enough	11 (33.3)
Other interventions avoided	6 (18.2)
High adherence	3 (9.1)
ITT^a^ analysis	3 (9.1)
Participants and providers blinded	2 (6.1)
Analyst blinded	1 (3.0)

a ITT: intention-to-treat.

**Table 5.** Numbers of studies without a control group that met each assessment item.

Assessment item	Value, n (%)
Objective clearly stated	10 (100.0)
Eligibility criteria clear	10 (100.0)
Intervention clear and consistent	10 (100.0)
Outcomes assessment	10 (100.0)
Analyze changes with *P* value	10 (100.0)
Follow-up rate	5 (50.0)
Outcomes measured multiple times	3 (30.0)
Participants representative	2 (20.0)
All eligible participants enrolled	1 (10.0)

## Discussion

### Principal Findings

This is the first publication to describe intervention studies focused on weight loss or obesity control conducted via WeChat. We identified gaps in the current design and implementation of these digital intervention studies, such as inadequate tracking options and social support, lack of individualized goals, and absence of health care experts [33]. Overall, the results are promising, suggesting that WeChat may be an effective platform for supporting weight loss or obesity management. However, more rigorous, randomized controlled trials are needed to understand the effectiveness of interventions delivered using this platform, and to determine the best implementation strategies. Our research revealed several important findings.

### Deficiency of Current Obesity Interventions via WeChat

None of the studies reported using formative research to design the intervention. Formative research is a process of collecting useful data about the targeted population and context before or during intervention implementation, during which behavioral change strategies are developed and refined to be maximally suitable [88]. This process helps with cultural contextualization and optimizes the impact of intervention strategies [89] by generating more effective interventions tailored to specific populations. For example, formative research on diabetes management and support indicated that intervention strategies for type 1 diabetes should be different from those for type 2 [90]. Thus, to better use WeChat in obesity intervention studies, formative research is imperative for developing further strategies to achieve weight management outcomes. Of the 6 studies [47,51,57,61,78,85] that reported no significant changes in obesity-related measures, all had a duration of less than 6 months. Intervention durations of 6-12 months and higher contact hours were correlated with more substantial changes [91,92]. Thus, future studies should consider extending intervention duration and enhance contact hours.

According to the assessment of studies using the Quality Assessment Tool designed by NIH [44], most studies had insufficient sample sizes. The intervention studies conducted in Chinese populations also had several common limitations. First, although most studies used randomization to assign participants to groups, blinding was rarely used (more than 95% did not report blinding during the intervention process). Similarly, information about participant adherence to interventions was limited, which impeded evaluation of effectiveness. In addition, our review of the inclusion and exclusion criteria found that most studies did not control for confounding effects caused by simultaneous participation in other intervention studies. These limitations might cast doubt on the generalizability of the findings.

### Majority WeChat Functions Used in Current Research

WeChat, one of the most widely used social media platforms in China, has many functions that can facilitate obesity interventions [93,94]. In recent years, more intervention studies have used WeChat as a tool in China [41,93-95] because of its convenience and wide reach. The Official Account and WeChat group were the most common features used in the intervention trials included in this review. Both one-to-one chatting and WeChat groups facilitated immediate communication. Although this type of immediate communication might occupy researchers’ time, it can also provide quick assistance to participants, potentially reducing loss to follow-up and increasing adherence to interventions, which may lead to positive outcomes. Official Account and Mini Program are user-friendly ways to disseminate intervention-related information, such as health knowledge and PA courses, as well as to record and evaluate personal data on daily diet and PA. While these functions could help researchers deliver health-related information, they do not ensure that participants actually engage with this information.

Generally, WeChat platform shows promise for obesity interventions; however, its utilization for health promotion still needs to be optimized. For example, although researchers can send intervention materials to participants easily via WeChat, there is no guarantee that they will pay attention to the health messages received [75]. Thus, study coordinators should not only use WeChat to deliver health-related information but also try to improve participant engagement through compelling content and strategies to compete for attention with the vast amount of the information disseminated through digital platforms [75].

### Future Directions—Use of WeChat in Future Obesity Intervention Research

#### Online Payment

To date, no study has incorporated the online payment function of WeChat into existing obesity interventions. A potential strategy could involve offering time-limited discounts on healthy foods (such as vegetables and fruits) to incentivize participants. Financial incentives have been shown to promote participants’ adherence and enhance the effectiveness of interventions [96]. Studies targeting obesity or overweight populations for weight loss indicated that financial incentives can motivate healthy behaviors and facilitate weight loss [97-99].

#### Living Streaming

WeChat’s live streaming function could encourage better engagement during health education. Because of the widespread transmission of the COVID-19 pandemic, people were required to quarantine at home and public places, such as parks and gyms, were closed, resulting in physical inactivity [100]. In view of this situation, the Chinese government expressed encouragement and support for live-streaming fitness in its National Fitness Plan (2021‐2025) [101]. Genghong Liu’s (a Taiwanese singer) free live-streamed exercise sessions on Douyin (the Chinese version of TikTok) attracted more than 13.9 billion viewers on April 17, 2022 [102]. This demonstrates the potential opportunity and serves as inspiration for using WeChat’s live-streaming function to provide guidance on weight control.

#### Peer Support

Based on our review of intervention strategies in the included studies, WeChat group function served as a platform on which participants could communicate with each other to exchange experience and thoughts and provide peer support as well as encouragement [49,56,63,73,86]. Group support in a study using Noom, an app for obesity intervention, demonstrated positive impacts of peer support within the group, including enhanced health education acquisition (such as reading and responding to health-themed information) and increased responses to others’ posts [103]. Evidence from Noom highlights the importance of social support, especially peer support, for future WeChat intervention studies.

#### Target Population

Although WeChat users are very diverse, older adults and young people, individuals with low education levels, and those living in poverty-stricken areas, many of whom are nonnetizens, still face difficulties using multiple WeChat functions or even accessing the platform [16]. Thus, implementing WeChat-based interventions among these populations might be limited.

### Limitations

Our review has several limitations. First, methodological heterogeneity and variable reporting quality in Chinese language studies limited the direct comparability of outcomes, but we addressed this using narrative synthesis by study type (with or without a control group). Second, while the cultural specificity of WeChat-based interventions limits generalizability, this review lays important groundwork for cross-platform comparisons.

### Conclusions

In summary, WeChat is a potential platform for delivering obesity interventions due to its wide use and various functions. Most of the reviewed papers reported effectiveness in terms of weight loss. However, intervention studies conducted via WeChat were limited in design and evaluation rigor. Therefore, future studies should include formative research for intervention development, use a randomized controlled blinded design, and use objective obesity outcome measures to increase the reliability of their findings. The main features of WeChat used in the included obesity interventions enabled chatting and information sharing without spatial limitation. Thus, further studies should explore multiple functions simultaneously to enhance participant engagement with health education materials, examine the impact of varying exposure levels, and increase participant engagement.

## Supplementary material

10.2196/65538 Multimedia Appendix 1 Search terms and quality assessment.

10.2196/65538 Checklist 1 PRISMA-ScR checklist.

## Abbreviations

BF: body fat
BFP: body fat percentage
CNKI: China National Knowledge Infrastructure
HC: hip circumference
mHealth: mobile health
NCD: noncommunicable chronic diseases
NIH: National Institutes of Health
NR: not report
PA: physical activity
PRISMA: Preferred Reporting Items for Systematic Reviews and Meta-Analyses
WC: waist circumference
WHR: waist-to-hip ratio

## References

[1] Congdon P (2019). Obesity and urban environments. Int J Environ Res Public Health.

[2] Lee EY, Yoon KH (2018). Epidemic obesity in children and adolescents: risk factors and prevention. Front Med.

[3] NHC (2020). Report on Chinese residents’ chronic diseases and nutrition 2020. Ying Yang Xue Bao.

[4] Chen K, Shen Z, Gu W (2023). Prevalence of obesity and associated complications in China: a cross-sectional, real-world study in 15.8 million adults. Diabetes Obes Metab.

[5] (2019). Global health data exchange. https://vizhub.healthdata.org/gbd-results?params=gbd-api-2019-permalink/dd64ad7d9c3fb70c835cbe706bc3fd9d.

[6] Wang Y, Zhao L, Gao L, Pan A, Xue H (2021). Health policy and public health implications of obesity in China. Lancet Diabetes Endocrinol.

[7] (2019). Healthy china initiative (2019–2030). National Health Commission of the People’s Republic of China.

[8] Roberto CA, Swinburn B, Hawkes C (2015). Patchy progress on obesity prevention: emerging examples, entrenched barriers, and new thinking. Lancet.

[9] Hall KD, Kahan S (2018). Maintenance of lost weight and long-term management of obesity. Med Clin North Am.

[10] Perdomo CM, Cohen RV, Sumithran P, Clément K, Frühbeck G (2023). Contemporary medical, device, and surgical therapies for obesity in adults. Lancet.

[11] Dumas AA, Lapointe A, Desroches S (2018). Users, uses, and effects of social media in dietetic practice: scoping review of the quantitative and qualitative evidence. J Med Internet Res.

[12] Bardus M, Smith JR, Samaha L, Abraham C (2015). Mobile phone and Web 2.0 technologies for weight management: a systematic scoping review. J Med Internet Res.

[13] Tully L, Sorensen J, O’Malley G (2021). Pediatric weight management through mHealth compared to face-to-face care: cost analysis of a randomized control trial. JMIR Mhealth Uhealth.

[14] Woo Baidal JA, Chang J, Hulse E, Turetsky R, Parkinson K, Rausch JC (2020). Zooming toward a telehealth solution for vulnerable children with obesity during Coronavirus disease 2019. Obesity (Silver Spring).

[15] Robbins R, Krebs P, Jagannathan R, Jean-Louis G, Duncan DT (2017). Health app use among US mobile phone users: analysis of trends by chronic disease status. JMIR Mhealth Uhealth.

[16] (2024). The 53rd China Statistical Report on internet development. https://cnnic.cn/NMediaFile/2024/0325/MAIN1711355296414FIQ9XKZV63.pdf.

[17] Ji X, Hu L, Wang Y (2019). “Mobile Health” for the management of spondyloarthritis and its application in China. Curr Rheumatol Rep.

[18] Mira JJ, Navarro I, Botella F (2014). A Spanish pillbox app for elderly patients taking multiple medications: randomized controlled trial. J Med Internet Res.

[19] Siriwoen R, Chongsuwat R, Tansakul S, Siri S (2018). Effectiveness of a weight management program applying mobile health technology as a supporting tool for overweight and obese working women. Asia Pac J Public Health.

[20] Toro-Ramos T, Lee DH, Kim Y (2017). Effectiveness of a smartphone application for the management of metabolic syndrome components focusing on weight loss: a preliminary study. Metab Syndr Relat Disord.

[21] Wang E, Abrahamson K, Liu PJ, Ahmed A (2020). Can mobile technology improve weight loss in overweight adults? A systematic review. West J Nurs Res.

[22] Haas K, Hayoz S, Maurer-Wiesner S (2019). Effectiveness and feasibility of a remote lifestyle intervention by dietitians for overweight and obese adults: pilot study. JMIR Mhealth Uhealth.

[23] McDiarmid S, Harvie M, Johnson R (2022). Manchester Intermittent versus Daily Diet App Study ( MIDDAS ): a pilot randomized controlled trial in patients with type 2 diabetes. Diabetes Obes Metab.

[24] Huntriss R, Haines M, Jones L, Mulligan D (2021). A service evaluation exploring the effectiveness of a locally commissioned tier 3 weight management programme offering face‐to‐face, telephone and digital dietetic support. Clin Obes.

[25] Beleigoli AM, Andrade AQ, Cançado AG, Paulo MN, Diniz M, Ribeiro AL (2019). Web-based digital health interventions for weight loss and lifestyle habit changes in overweight and obese adults: systematic review and meta-analysis. J Med Internet Res.

[26] Cueto V, Wang CJ, Sanders LM (2019). Impact of a mobile app-based health coaching and behavior change program on participant engagement and weight status of overweight and obese children: retrospective cohort study. JMIR Mhealth Uhealth.

[27] Chew CSE, Davis C, Lim JKE (2021). Use of a mobile lifestyle intervention app as an early intervention for adolescents with obesity: single-cohort study. J Med Internet Res.

[28] Jelalian E, Darling K, Foster GD, Runyan T, Cardel MI (2023). Effectiveness of a scalable mHealth intervention for children with overweight and obesity. Child Obes.

[29] Vidmar AP, Salvy SJ, Wee CP (2023). An addiction-based digital weight loss intervention: a multi-centre randomized controlled trial. Pediatr Obes.

[30] Wang Y, Willis E (2016). Examining theory-based behavior-change constructs, social interaction, and sociability features of the weight watchers’ online community. Health Educ Behav.

[31] Thomas JG, Raynor HA, Bond DS (2017). Weight loss in Weight Watchers Online with and without an activity tracking device compared to control: a randomized trial. Obesity (Silver Spring).

[32] Godino JG, Merchant G, Norman GJ (2016). Using social and mobile tools for weight loss in overweight and obese young adults (Project SMART): a 2 year, parallel-group, randomised, controlled trial. Lancet Diabetes Endocrinol.

[33] Rivera J, McPherson A, Hamilton J (2016). Mobile apps for weight management: a scoping review. JMIR Mhealth Uhealth.

[34] (2024). Tencent announces 2024 first quarter results. Tencent.

[35] Liu J, Zheng X, Zhang X, Feng Z, Song M, Lopez V (2020). The evidence and future potential of WeChat in Providing support for Chinese parents of pediatric patients undergoing herniorrhaphy. J Transcult Nurs.

[36] Li T, Ding W, Li X, Lin A (2019). Mobile health technology (WeChat) for the hierarchical management of community hypertension: protocol for a cluster randomized controlled trial. Patient Prefer Adherence.

[37] Li WW, Toh P (2023). WeChat-based intervention for Chinese immigrants with hypertension: development and evaluation study. Asian Pac Isl Nurs J.

[38] Wang Y, Guo F, Wang J (2023). Efficacy of a WeChat-based multimodal digital transformation management model in new-onset mild to moderate hypertension: randomized clinical trial. J Med Internet Res.

[39] Dong Y, Wang P, Dai Z (2018). Increased self-care activities and glycemic control rate in relation to health education via Wechat among diabetes patients: a randomized clinical trial. Medicine (Baltimore).

[40] Ding B, Gou B, Guan H, Wang J, Bi Y, Hong Z (2021). WeChat-assisted dietary and exercise intervention for prevention of gestational diabetes mellitus in overweight/obese pregnant women: a two-arm randomized clinical trial. Arch Gynecol Obstet.

[41] Tian Y, Zhang S, Huang F, Ma L (2021). Comparing the efficacies of telemedicine and standard prenatal care on blood glucose control in women with gestational diabetes mellitus: randomized controlled trial. JMIR Mhealth Uhealth.

[42] Yang J, Yang H, Wang Z (2021). Self-management among type 2 diabetes patients via the WeChat application: a systematic review and meta-analysis. J Clin Pharm Ther.

[43] Page MJ, McKenzie JE, Bossuyt PM (2021). The PRISMA 2020 statement: an updated guideline for reporting systematic reviews. Syst Rev.

[44] (2013). Study quality assessment tools. National Heart, Lung, and Blood Institute.

[45] Xia SF, Maitiniyazi G, Chen Y (2022). Web-based TangPlan and WeChat combination to support self-management for patients with type 2 diabetes: randomized controlled trial. JMIR Mhealth Uhealth.

[46] He C, Wu S, Zhao Y (2017). Social media-promoted weight loss among an occupational population: cohort study using a WeChat mobile phone app-based campaign. J Med Internet Res.

[47] Chen JL, Guo J, Zhong Q (2023). Smartphone-Based Cancer and Obesity Prevention Education Program for Chinese Women (SCOPE): a pilot RCT. Int J Environ Res Public Health.

[48] Liu Y, Sun P, Shuai P, Qiao Q, Li T (2021). Fat-restricted low-glycemic index diet controls weight and improves blood lipid profile: a pilot study among overweight and obese adults in Southwest China. Medicine (Baltimore).

[49] Li J, Yang H, Song X (2023). Effectiveness of social media with or without wearable devices to improve physical activity and reduce sedentary behavior: a randomized controlled trial of Chinese postgraduates. Heliyon.

[50] Han Y, Ye X, Li X (2023). Comparison of an online versus conventional multidisciplinary collaborative weight loss programme in type 2 diabetes mellitus: a randomized controlled trial. Int J Nurs Pract.

[51] Ling D, Wang R, Chen Q (2021). Assessment of chronic disease management mode (CDMM) on participants with primary hypertension. Trop Med Int Health.

[52] Yang X, Chattopadhyay K, Hubbard R, Li JL, Li L, Lin Y (2021). 36-Month evaluation of a weight management programme in Chinese overweight and obese adults. Front Public Health.

[53] Linjuan Z (2023). Analysis of risk factors of cardiovascular disease in type 2 diabetes patients and intervention of intelligent remote blood glucose management [Master’s thesis]. https://link.oversea.cnki.net/doi/10.27231/d.cnki.gnmyc.2023.000231.

[54] Zihao P, Qi W, Mu C (2022). Analysis of weight loss effect of personalized high protein dietary intervention in overweight/obese people. Appl Prev Med.

[55] Xi C, Juan H, Hong L (2021). The application of 5+2 intermittent fasting in weight loss of employees in an affiliated hospital of a medical university. Med Diet Health.

[56] Dongmei X, Hui L, Danfeng C (2016). Application of Internet in health education for patients with coronary heart disease. J Qilu Nurs.

[57] Bei H (2018). The application of mobile health treatment based on “internet +” for the health management of hypertension patients in a community [Master’s thesis]. https://oversea.cnki.net/KCMS/detail/detail.aspx?dbcode=CMFD&dbname=CMFD201802&filename=1018212801.nh&uniplatform=OVERSEA&v=y_mpMf-c8AC-34R5kOgBNFkNgMHjc6We4usAY6waBSXKjhvIaEoNdgxGyD0Iemq2.

[58] Xiaoyuan H, Meirong Z, Zhifang T (2022). Application of traditional Chinese medicine dietary therapy combined with WeChat exercise in nursing of coronary heart disease patients with phlegm dampness constitution. Dang Dai Hu Shi.

[59] Jinhua Y (2022). Based on multi-theory model the study on comprehensive intervention of behavior changes for elderly patients with hypertension [phd thesis]. https://link.oversea.cnki.net/doi/10.27138/d.cnki.ghuzc.2022.000074.

[60] Jiawei C (2020). Clinical study on the influence of traditional chinese medicine behavior intervention and health management on the quality of life of type 2 diabetes patients [Master’s thesis]. https://link.oversea.cnki.net/doi/10.26988/d.cnki.gcdzu.2020.000194.

[61] Yuepeng T (2021). A comparative study of online and offline exercise health education on behavioral changes in patients with hyperuricemia [Master’s thesis]. https://link.oversea.cnki.net/doi/10.27364/d.cnki.gttyy.2021.000133.

[62] Li Y (2018). Design and application of internet plus nutrition and weight management system for diabetic patients [Master’s thesis]. https://link.oversea.cnki.net/doi/10.27001/d.cnki.gtjyu.2020.000291.

[63] Yingming M (2020). Effect of health belief model on weight management in overweight or obese polycystic ovary syndrome patients with infertility [Master’s thesis]. https://link.oversea.cnki.net/doi/10.27952/d.cnki.ghnyx.2020.000046.

[64] Xiaojuan Y, Qun L, Mingzhu C (2018). Effect of individualized diet and exercises based on the WeChat on the overweight population with prediabetes. Nurs Integr Tradit Chin West Med.

[65] Peijun H (2022). Effect of traditional Chinese medicine health care guidance on the WeChat platform on body weight and quality of life for patients with obesity. J Sichuan Tradit Chin Med.

[66] Yuexi Y, Yanxia L, Xiao W (2019). Effect of WeChat multimedia platform on the glucose metabolism in pre diabetes population. J SNAKE Sci Nat.

[67] Jiangping M, Qiuli Z, Shanshan C (2016). The effectiveness of exercise intervention on exercise adherence among college teachers with dyslipidemia. Chin J Nurs Educ.

[68] Xiao Z, Dafang C (2019). Effects of lifestyle intervention by internet-based online on weight loss in people with weight loss willingness. Chin J Prev Contr Chron Dis.

[69] Tianmeng R, Jie R, Zilong L (2023). Effects of short-term walking intervention on physical health indicators in occupational population with higher blood pressure. Chin J Prev Contr Chron Dis.

[70] Ziwei Y (2021). Effects of wechat intervention based on BCW+bcts on the treatment compliance of young and middle-aged patients with hypertension [Master’s thesis]. https://link.oversea.cnki.net/doi/10.27234/d.cnki.gnhuu.2021.000792.

[71] Jianxin M, Jinping Z, Lian C (2020). Efficiency of health management for elderly hypertensive patients using WeChat Applet. Chin J Mult Organ Dis Elderly.

[72] Di P (2017). Energy balance and imbalance of obese children’s weight changes—an intervention program on diet and exercise [phd thesis]. https://oversea.cnki.net/KCMS/detail/detail.aspx?dbcode=CDFD&amp;dbname=CDFDLAST2017&amp;filename=1017181664.nh&amp;uniplatform=OVERSEA&amp;v=KgfQHfMuKQK2t_YxPsLSMI5ih1thlN_qdktKt8v4-MYCTcBCi7dVsvMwGkS0A-Ep.

[73] Ruixue B, Xiaoya Q, Yukun H (2023). Evaluation of mobile application-based physician active management on weight loss in overweight and obese patients. Health Med Res Prac.

[74] Youxuan Y (2023). Evaluation of the effect of internet plus intervention on blood pressure control in high risk population of hypertension in haikou community [master’s thesis]. https://oversea.cnki.net/KCMS/detail/detail.aspx?dbcode=CMFD&dbname=CMFD202401&filename=1023643307.nh&uniplatform=OVERSEA&v=aTZXVkrghMBTfdvmOY4tHZLSk_u6Qm2KuTOkmTBO3dSsMo6pPUO-u2ulv3szPLG9.

[75] Jielili M (2022). Evaluation of the weight loss effect of whole wheat flour on obese people [Master’s thesis]. https://link.oversea.cnki.net/doi/10.27433/d.cnki.gxyku.2020.000396.

[76] Mengqin W (2023). Health management and its evaluation of essential hypertension among residents in underdeveloped rural areas of shanxi province [Master’ thesis]. https://oversea.cnki.net/KCMS/detail/detail.aspx?dbcode=CMFD&dbname=CMFD202401&filename=1023825479.nh&uniplatform=OVERSEA&v=EYMG9C7Wn20OxkBNdrwiNNz_kZKLad7v2IaZeI-njwMio3ChEKPxbbFEWt7or1wQ.

[77] Xujuan M, Junfeng Z, Minghou Z (2019). The impact of 8-week health management on fat mass and other indicators in obesity population. J Shanxi Med Coll Contin Educ.

[78] Wei Y, Yongyan Y, Xiaokang H (2023). The impact of health education on WeChat platform on the health knowledge and behavior of college students with normal high blood pressure. Xizang Med.

[79] Rongrong L (2019). Management of patients with prediabetes based on wechat platform and its effect analysis [Master’s thesis]. https://link.oversea.cnki.net/doi/10.27433/d.cnki.gxyku.2019.000060.

[80] Huirong G, Xueqin W, Wei L (2017). Research of intervention by “public health microworld” WeChat platform for lifestyle. China Prac Med.

[81] Weiwei C (2022). Research on the intervention effect of aerobic exercise program on the physical inactivity of the elderly patients with chronic diseases based on COM-b model [Master’s thesis]. https://link.oversea.cnki.net/doi/10.26925/d.cnki.gbbyc.2022.000289.

[82] Moufu W, Xuejiao Z, Ting L (2022). Research on the management effect of “Internet +” self-management mode on diabetes mellitus patients. China Prac Med.

[83] Lu Q (2023). Study on the application of calorie restrict diet intervention based on IMB model in young and middle-aged patients with coronary heart disease [master’s thesis]. https://link.oversea.cnki.net/doi/10.27108/d.cnki.ghelu.2023.000895.

[84] Yinbao S (2023). Study on the effect of health education on cardiovascular diseases among Uyghur population in rural areas of Xinjiang production and construction corps [Master’s thesis]. https://oversea.cnki.net/KCMS/detail/detail.aspx?dbcode=CMFD&dbname=CMFD202401&filename=1023708617.nh&uniplatform=OVERSEA&v=DfrWkfZQKBjDQeEDJu77c2GIA4DmAsqAcAOKzlc7kL0GTwVfntpWdPfkwrI-E7DB.

[85] Lijuan Z (2020). Study on the evaluation of intervention effects of the O2O-based community management model for young and middle-aged hypertension patients [master’s thesis]. https://link.oversea.cnki.net/doi/10.27020/d.cnki.gfjyu.2020.000195.

[86] Jun L (2023). Study on the intervention effect of eight- form tai chi on pre-diabetic people under different exercise management mode [Master’s thesis]. https://oversea.cnki.net/KCMS/detail/detail.aspx?dbcode=CMFD&amp;dbname=CMFD202401&amp;filename=1023765390.nh&amp;uniplatform=OVERSEA&amp;v=W1Vtw8Qsgx4EWVhrd4mflQb1RkwPYXr7mWjbCKHCKylvMcYgJhL9MJGJ_Ui9opnP.

[87] Jingxia K (2020). Worksite-based intervention on prevention and control of overweight and obesity [phd thesis]. https://oversea.cnki.net/KCMS/detail/detail.aspx?dbcode=CDFD&dbname=CDFDLAST2021&filename=1020435505.nh&uniplatform=OVERSEA&v=CexL3PgZ7pMiYuDn4f68lVAyrrJMoMdLTzzVAfAEE1UX0mb2H9BVIzVX11AfBpMt.

[88] Gittelsohn J, Steckler A, Johnson CC (2006). Formative research in school and community-based health programs and studies: “state of the art” and the TAAG approach. Health Educ Behav.

[89] Bentley ME, Johnson SL, Wasser H (2014). Formative research methods for designing culturally appropriate, integrated child nutrition and development interventions: an overview. Ann N Y Acad Sci.

[90] Gupta L, Lal PR, Gupta Y, Goyal A, Khanna A, Tandon N (2021). Formative research to develop diabetes self-management education and support (DSMES) program for adults with type 1 diabetes. Diabetes Metab Syndr.

[91] Adebile TV, Adebile TM, Oloyede TF (2025). Telemedicine for obesity management among United States adults: a systematic and meta-analysis of intervention studies. J Telemed Telecare.

[92] James A, Afable A, Bayoumi N, Dhuper S (2023). Evaluation of a childhood obesity program serving a high-need population in Brooklyn, New York using survival analysis. Int J Environ Res Public Health.

[93] Yun K, Chu Z, Zhang J (2021). Mobile phone intervention based on an HIV risk prediction tool for HIV prevention among men who have sex with men in China: randomized controlled trial. JMIR Mhealth Uhealth.

[94] Shangguan F, Wang R, Quan X (2021). Association of stress-related factors with anxiety among Chinese pregnant participants in an online crisis intervention during COVID-19 epidemic. Front Psychol.

[95] Song Y, Xie X, Chen Y (2021). The effects of WeChat-based educational intervention in patients with ankylosing spondylitis: a randomized controlled trail. Arthritis Res Ther.

[96] Voils CI, Pendergast J, Hale SL (2021). A randomized feasibility pilot trial of a financial incentives intervention for dietary self-monitoring and weight loss in adults with obesity. Transl Behav Med.

[97] Agarwal AK, Waddell KJ, Small DS (2021). Effect of gamification with and without financial incentives to increase physical activity among veterans classified as having obesity or overweight: a randomized clinical trial. JAMA Netw Open.

[98] Hoddinott P, O’Dolan C, Macaulay L (2024). Text messages with financial incentives for men with obesity: a randomized clinical trial. JAMA.

[99] Yeung KF, Gandhi M, Lam AYR (2021). The Pre-Diabetes Interventions and Continued Tracking to Ease-out Diabetes (Pre-DICTED) program: study protocol for a randomized controlled trial. Trials.

[100] Tian R, Yin R, Gan F (2022). Exploring public attitudes toward live-streaming fitness in China: a sentiment and content analysis of China’s social media Weibo. Front Public Health.

[101] (2021). The National Fitness Plan (2021-2025). The State Council of the People’s Republic of China.

[102] (2022). Liu Genghong, dubbed “next Li Jiaqi”, unlocks China’s fitness live stream. Dao Insights.

[103] Kim H, Ray CD, Veluscek AM (2017). Complementary support from facilitators and peers for promoting mHealth engagement and weight loss. J Health Commun.

