# Supplementary material for: Obesity Prevention and Reduction in China Using the Social Media Platform WeChat: Scoping Review
Source: Interact J Med Res. 2025 Dec 11;14:e65538. doi: 10.2196/65538 (PMC12698035; doi:10.2196/65538)
Supplement: Multimedia Appendix 1 [file ijmr-v14-e65538-s001.docx]

Table S1. Search terms used in both English and Chinese language databases.

| **Database** | **Search terms** |
| --- | --- |
| Pubmed | ((obesity[Title/Abstract]) OR (weight[Title/Abstract]) OR (BMI[Title/Abstract]) OR (waist circumference[Title/Abstract]) OR (hip circumference[Title/Abstract]) OR (waist to hip ratio[Title/Abstract]) OR (body fat[Title/Abstract]) OR (skin fold thickness[Title/Abstract])) AND (WeChat[Title/Abstract]) |
| CNKI | ((feipang[Title/Keywords/Abstract]) OR (tizhong[Title/Keywords/Abstract]) OR (tizhongzhishu[Title/Keywords/Abstract] OR (yaowei[Title/Keywords/Abstract]) OR (tunwei[Title/Keywords/Abstract]) OR (yaotunbi[Title/Keywords/Abstract]) OR (tizhi[Title/Keywords/Abstract]) OR (pizhehoudu[Title/Keywords/Abstract])) AND (weixin[Title/Keywords/Abstract]) |

Table S2. Quality assessment of controlled-intervention studies ^a^ (in order of publication date).

|  | 1 | 2 | 3 | 4 | 5 | 6 | 7 | 8 | 9 | 10 | 11 | 12 | 13 | 14 | Rating^b^ |
| --- | --- | --- | --- | --- | --- | --- | --- | --- | --- | --- | --- | --- | --- | --- | --- |
| Li et al [49] | ✓ | NR | NR | ✗ | NR | NR | ✓ | ✓ | NR | NR | ✓ | ✓ | ✓ | ✗ | F |
| Chen et al [47] | ✓ | NR | NR | NR | NR | ✓ | ✓ | ✓ | ✓ | NR | ✓ | ✗ | ✓ | ✓ | F |
| Han et al [50] | ✓ | ✓ | ✓ | ✓ | ✓ | ✓ | ✓ | ✓ | NR | ✓ | ✓ | ✗ | ✓ | ✓ | G |
| Xia et al [45] | ✓ | NR | NR | ✗ | ✗ | ✓ | ✗ | ✓ | NR | NR | ✓ | ✓ | ✓ | ✗ | F |
| Ling et al [51] | ✓ | ✓ | NR | ✗ | NR | ✓ | ✓ | ✓ | NR | NR | ✓ | NR | ✓ | NA | F |
| He et al [46] | ✗ | ✗ | ✗ | NR | NR | ✗ | ✓ | ✓ | ✗ | NR | ✓ | NR | ✓ | ✗ | P |
| Yinbao [84] | ✓ | ✗ | ✗ | NR | NR | ✓ | ✓ | ✓ | NR | NR | ✓ | ✓ | ✓ | ✗ | F |
| Lu [83] | ✓ | ✓ | ✓ | NR | NR | ✓ | ✓ | ✓ | NR | NR | ✓ | ✓ | ✓ | ✗ | F |
| Youxuan [74] | ✓ | ✓ | ✓ | NR | NR | ✓ | ✓ | ✓ | NR | NR | ✓ | ✓ | ✓ | NR | F |
| Jun[86] | ✓ | NR | NR | NR | NR | NR | ✓ | ✓ | ✓ | NR | ✓ | ✗ | ✓ | ✗ | F |
| Mengqin [76] | ✓ | ✓ | ✓ | ✓ | NR | ✓ | ✓ | ✓ | NR | NR | ✓ | ✓ | ✓ | ✗ | G |
| Wei et al [78] | ✓ | NR | NR | NR | NR | NR | ✓ | ✓ | NR | NR | ✓ | NR | ✓ | NA | F |
| Moufu et al [82] | ✓ | NR | NR | NR | NR | NR | ✓ | ✓ | NR | NR | ✓ | NR | ✓ | NA | F |
| Xiaoyuan et al [58] | ✓ | ✓ | ✓ | NR | NR | ✓ | ✓ | ✓ | NR | NR | ✓ | ✗ | ✓ | ✗ | F |
| Weiwei [81] | ✗ | ✗ | ✗ | ✗ | NR | ✓ | ✓ | ✓ | NR | ✓ | ✓ | ✗ | ✓ | ✗ | F |
| Jinhua [59] | ✓ | ✓ | ✓ | NR | NR | ✓ | ✓ | ✓ | NR | NR | ✓ | ✓ | ✓ | ✗ | F |
| Peijun [65] | ✓ | ✓ | ✓ | NR | NR | ✓ | NR | NR | NR | NR | ✓ | NR | ✓ | NR | F |

1. Was the study described as randomized, a randomized trial, a randomized clinical trial, or an RCT?

2. Was the method of randomization adequate (i.e., use of randomly generated assignment)?

3. Was the treatment allocation concealed (so that assignments could not be predicted)?

4. Were study participants and providers blinded to treatment group assignment?

5. Were the people assessing the outcomes blinded to the participants' group assignments?

6. Were the groups similar at baseline on important characteristics that could affect outcomes (e.g., demographics, risk factors, co-morbid conditions)?

7. Was the overall drop-out rate from the study at endpoint 20% or lower of the number allocated to treatment?

8. Was the differential drop-out rate (between treatment groups) at endpoint 15 percentage points or lower?

9. Was there high adherence to the intervention protocols for each treatment group?

10. Were other interventions avoided or similar in the groups (e.g., similar background treatments)?

11. Were outcomes assessed using valid and reliable measures, implemented consistently across all study participants?

12. Did the authors report that the sample size was sufficiently large to be able to detect a difference in the main outcome between groups with at least 80% power?

13. Were outcomes reported or subgroups analyzed prespecified (i.e., identified before analyses were conducted)?

14. Were all randomized participants analyzed in the group to which they were originally assigned, i.e., did they use an intention-to-treat analysis?

^a^ Abbreviations: CD, cannot determine; NA, not applicable; NR, not reported. ^b^ Rating: G, good; F, fair; P, poor.

Rater 1 :YNW; Rater 2: XXZ.

Table S2. Quality assessment of controlled-intervention studies ^a^ (in order of publication date) (Continued)

|  | 1 | 2 | 3 | 4 | 5 | 6 | 7 | 8 | 9 | 10 | 11 | 12 | 13 | 14 | Rating^b^ |
| --- | --- | --- | --- | --- | --- | --- | --- | --- | --- | --- | --- | --- | --- | --- | --- |
| Jielili [75] | ✓ | NR | ✗ | NR | NR | ✗ | NR | NR | NR | NR | ✓ | NR | ✓ | NR | P |
| Ziwei [70] | ✓ | ✓ | ✓ | NR | NR | ✓ | ✓ | ✓ | NR | ✓ | ✓ | ✗ | ✓ | NA | G |
| Yuepeng [61] | ✓ | NR | NR | NR | NR | ✓ | ✓ | ✓ | ✗ | NR | ✓ | NR | ✓ | NA | F |
| Jianxin et al [71] | ✗ | ✗ | ✗ | NR | NR | ✓ | ✓ | ✓ | NR | NR | ✓ | NR | ✓ | NA | F |
| Li [62] | ✓ | ✓ | ✓ | NR | NR | ✓ | ✓ | ✓ | NR | NR | ✓ | ✗ | ✓ | ✗ | F |
| Yingming [63] | ✓ | ✗ | ✗ | NR | NR | ✓ | ✓ | ✓ | NR | NR | ✓ | ✓ | ✓ | ✗ | F |
| Lijuan [85] | ✓ | ✓ | ✓ | NR | NR | ✓ | ✓ | ✓ | NR | NR | ✓ | ✗ | ✓ | ✗ | F |
| Jiawei [60] | ✓ | ✓ | ✓ | NR | NR | ✓ | ✓ | ✓ | NR | ✓ | ✓ | ✗ | ✓ | ✗ | F |
| Yuexi et al [66] | ✓ | ✓ | ✓ | NR | NR | ✗ | ✓ | ✓ | NR | NR | ✓ | ✗ | ✓ | NA | F |
| Jingxia et al [87] | ✗ | ✗ | ✗ | ✗ | NR | ✗ | ✗ | ✓ | ✗ | ✓ | ✓ | ✓ | ✓ | ✓ | F |
| Rongrong [79] | ✓ | ✓ | ✓ | NR | NR | ✓ | ✓ | ✓ | NR | ✓ | ✓ | ✓ | ✓ | ✗ | G |
| Bei [57] | ✓ | ✓ | ✓ | NR | NR | ✓ | ✓ | ✓ | NR | NR | ✓ | ✓ | ✓ | ✗ | F |
| Huirong et al [80] | ✗ | ✗ | ✗ | NR | NR | NR | ✓ | ✓ | NR | NR | ✓ | NR | ✓ | ✗ | P |
| Di [72] | ✓ | NR | NR | NR | NR | NR | NR | NR | ✓ | NR | ✓ | ✗ | ✓ | NR | P |
| Jiangping et al [67] | ✗ | ✗ | ✗ | NR | NR | ✓ | ✓ | ✓ | NR | NR | ✓ | ✗ | ✓ | ✗ | F |
| Dongmei et al [56] | ✓ | ✓ | ✓ | NR | NR | ✓ | NR | NR | NR | NR | ✓ | NR | ✓ | NR | F |

1. Was the study described as randomized, a randomized trial, a randomized clinical trial, or an RCT?

2. Was the method of randomization adequate (i.e., use of randomly generated assignment)?

3. Was the treatment allocation concealed (so that assignments could not be predicted)?

4. Were study participants and providers blinded to treatment group assignment?

5. Were the people assessing the outcomes blinded to the participants' group assignments?

6. Were the groups similar at baseline on important characteristics that could affect outcomes (e.g., demographics, risk factors, co-morbid conditions)?

7. Was the overall drop-out rate from the study at endpoint 20% or lower of the number allocated to treatment?

8. Was the differential drop-out rate (between treatment groups) at endpoint 15 percentage points or lower?

9. Was there high adherence to the intervention protocols for each treatment group?

10. Were other interventions avoided or similar in the groups (e.g., similar background treatments)?

11. Were outcomes assessed using valid and reliable measures, implemented consistently across all study participants?

12. Did the authors report that the sample size was sufficiently large to be able to detect a difference in the main outcome between groups with at least 80% power?

13. Were outcomes reported or subgroups analyzed prespecified (i.e., identified before analyses were conducted)?

14. Were all randomized participants analyzed in the group to which they were originally assigned, i.e., did they use an intention-to-treat analysis?

^a^ Abbreviations: NA, not applicable; NR, not reported. ^b^ Rating: G, good; F, fair; P, poor.

Rater 1 :YNW; Rater 2: XXZ.

Table S3. Quality assessment of before-after (pre-post) studies^a^ (in order of publication date).

|  | 1 | 2 | 3 | 4 | 5 | 6 | 7 | 9 | 10 | 11 | 12 | Rating^b^ |
| --- | --- | --- | --- | --- | --- | --- | --- | --- | --- | --- | --- | --- |
| Yang et al[52] | ✓ | ✓ | ✓ | ✗ | NR | ✓ | ✓ | ✗ | ✓ | ✓ | NA | F |
| Liu et al [48] | ✓ | ✓ | ✓ | ✗ | ✗ | ✓ | ✓ | ✓ | ✓ | ✗ | NA | F |
| Linjuan [53] | ✓ | ✓ | ✗ | ✗ | ✗ | ✓ | ✓ | ✗ | ✓ | ✗ | NA | F |
| Tianmeng et al [69] | ✓ | ✓ | ✗ | ✓ | NR | ✓ | ✓ | ✓ | ✓ | ✗ | NA | F |
| Ruixue et al [73] | ✓ | ✓ | ✗ | NR | ✗ | ✓ | ✓ | ✗ | ✓ | ✗ | NA | F |
| Zihao et al [54] | ✓ | ✓ | NR | NR | ✗ | ✓ | ✓ | ✓ | ✓ | ✗ | NA | F |
| Xi et al [55] | ✓ | ✓ | NR | ✗ | NR | ✓ | ✓ | ✓ | ✓ | ✗ | NA | F |
| Xujuan et al [77] | ✓ | ✓ | NR | NR | NR | ✓ | ✓ | NR | ✓ | ✗ | NA | F |
| Xiao et al[68] | ✓ | ✓ | NR | NR | NR | ✓ | ✓ | ✓ | ✓ | ✓ | NA | F |
| Xiaojuan et al [64] | ✓ | ✓ | NR | NR | ✗ | ✓ | ✓ | NR | ✓ | ✓ | NA | F |

1. Was the study question or objective clearly stated?

2. Were eligibility/selection criteria for the study population prespecified and clearly described?

3. Were the participants in the study representative of those who would be eligible for the test/service/intervention in the general or clinical population of interest?

4. Were all eligible participants that met the prespecified entry criteria enrolled?

5. Was the sample size sufficiently large to provide confidence in the findings?

6. Was the test/service/intervention clearly described and delivered consistently across the study population?

7. Were the outcome measures prespecified, clearly defined, valid, reliable, and assessed consistently across all study participants?

8. Were the people assessing the outcomes blinded to the participants’ exposures/interventions?

9. Was the loss to follow-up after baseline 20% or less? Were those lost to follow-up accounted for in the analysis?

10. Did the statistical methods examine changes in outcome measures from before to after the intervention? Were statistical tests done that provided p values for the pre-to-post changes?

11. Were outcome measures of interest taken multiple times before the intervention and multiple times after the intervention (i.e., did they use an interrupted time-series design)?

^a^ Abbreviations: NA, not applicable; NR, not reported. ^b^ Rating: G, good; F, fair; P, poor.

Rater 1 :YNW; Rater 2: XXZ.
